# Aneurysm of the Internal Carotid Artery Misdiagnosed as Cervical Paraganglioma; A Case Report and Literature Review

**DOI:** 10.29252/beat-070413

**Published:** 2019-10

**Authors:** Hossein Hodjati, Hamed Ghoddusi Johari, Bijan Khademi, Abdolkarim Rahmanian, Abtin Vahidi, Maryam Dehghankhalili

**Affiliations:** 1 *Department of General Surgery, Shiraz University of Medical Sciences, Shiraz, Iran*; 2 *Department of Otolaryngology, Otolaryngology Research Center, Shiraz University of Medical Sciences, Shiraz, Iran*; 3 *Department of Neurosurgery, Shiraz University of Medical Sciences, Shiraz, Iran*; 4 *Student Research Committee, Shiraz University of Medical Sciences, Shiraz, Iran *

**Keywords:** Aneurysm, Internal carotid artery, Extracranial segment, Surgical repair, Paraganglioma

## Abstract

The aneurysms of the extracranial segment of the internal carotid artery are not common and are associated with severe neurologic deficits. They could be misdiagnosed with several lesion of the cervical region. We herein report a case of internal carotid artery aneurysm misdiagnosed as paraganglioma. A 23-year-old man presented with progressive growing mass in right enlarging mass in the upper part of the neck below the angle of the mandible. The patient underwent surgery by the ear, nose, throat (ENT) surgeon through submandibular approach with impression of paraganglioma but severe pulsatile bleeding was encountered intraoperatively. Two vascular clamps were applied and the patient was transferred to the vascular ward. Computerized tomography (CT) angiogram revealed a huge aneurysm of the internal carotid artery in the extracranial segment with injured wall. After 2 days of medical therapy the patient was transferred to the operating room and the aneurysm was repaired using Dacrons. The patient had an uneventful hospital course and was asymptomatic after 1 year of follow-up. Precise preoperative assessment and evaluation with different modalities should be performed to avoid fatal complications. Surgery is a safe and effective method in experienced hands for repair of such aneurysms.

## Introduction

The aneurysms of the extracranial segment of the internal carotid arteries are nor very common but are considered an important clinical entity because of their association with high risk of neurological thromboembolic events, cranial nerve compression, and, more rarely, rupture [1, 2]. The etiology of extracranial aneurysms (ECA) includes atherosclerosis, dysplastic, infection and trauma [1, 3]. The most commonly involved artery is the internal carotid artery, but any segment of the carotid artery such as common, external and internal part can be affected [1]. The diagnosis of the ECA could be a dilemma to the vascular surgeons because of similar morphology and characteristics to several other pathologies in the region [4]. Due to this complexity, some reports of misdiagnosis of these aneurysms with other cervical pathologies exists in the English literature [5, 6]. We herein, report a case of unilateral extracranial segment of the internal carotid artery which was primarily misdiagnosed as paraganglioma and underwent a secondary surgery.

## Case Report

**Fig. 1:**
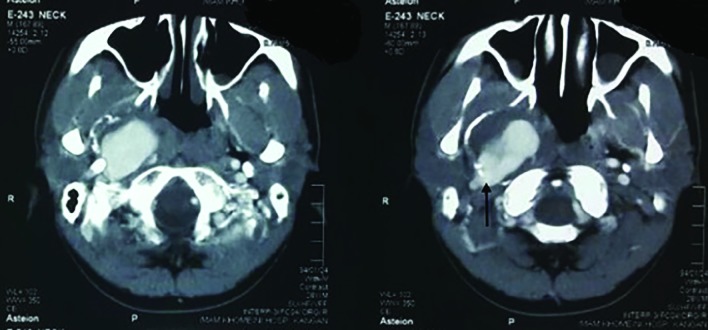
Axial computed tomography (CT) angiogram of the patient revealed a huge aneurysm at the distal segment of the internal carotid artery. The aneurysm is injured as demonstrated by extravasation of the contrast material (Arrow)

**Fig. 2:**
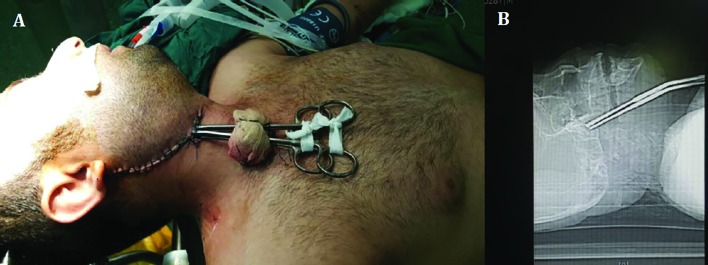
The intraoperative image of the patient demonstrating temporary closing of the wound with two vascular clamps in place for bleeding control (A); Scot image of the computed tomography (CT) scan of the patient demonstrating the clamps in place

A 23-year-old man presented to our outpatient clinic with painless gradually enlarging mass in the upper part of the neck below the angle of the mandible. Computed tomography (CT) scan of the lesion demonstrated homogenous, hyperdense, lesion at the mandibular angle which was diagnosed as aparaganglioma. The patient underwent surgery by the ear, nose, throat (ENT) surgeon through submandibular approach. After manipulation of the lesion, severe pulsatile bleeding was encountered and a vascular lesion was suspected. Thus two vascular clamps were placed proximal and distal to the lesion and the bleeding was controlled. Vascular surgeon was consulted immediately. A CT-angiogram revealed a huge (8×4 cm) aneurysm located at the distal part of the right internal carotid artery (ICA) aneurysm which was injured (Figure 1). Anticoagulation and wide spectrum antibiotic was administered to the patient and he was admitted to the intensive care unit (ICU) with the clamps in place (Figure 2). 

**Fig. 3:**
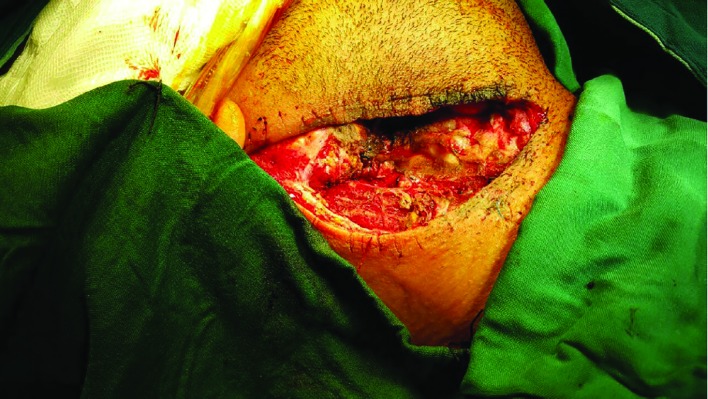
The intraoperative image demonstrating the aneurysm in distal segment of the internal carotid artery after reoperation

After 2 days with available neurovascular surgeon for possible intracranial bleeding control and craniotomy, the patient was transferred to operating room. The previous skin incision was re-opened and the two gauzes were removed (Figure 3). Appropriate irrigation was performed and the two vascular clamps were removed one by one. There was just small back bleeding from distal neck of right ICA aneurysm, therefore packing by means of small segments of fibrillar Dacron was done for 15 minutes and bleeding was stopped completely. Then Dacrons were covered with muscle flaps and the wound was closed in layers. The patient had an uneventful postoperative course and was discharged on the 6^th^ postoperative day. The postoperative angiogram revealed the patent internal carotid artery without any aneurysm residue. The patient received anticoagulation for 3 months and was followed in outpatient clinic. After 1 year of follow-up he was completely asymptomatic and the angiogram revealed patent parent vessel without residue. The patient and his family provided their informed written consent for publication of the case and the images. 

## Discussion

In the current study we have described a rare case of extracranial segment of the internal carotid artery misdiagnosed as paraganglioma. The patient underwent a first surgery for resection of the lesion but severe pulsatile bleeding stopped the operation and the second operation was performed after angiographic evaluation. The aneurysm was successfully repaired. Aneurysms can be categorized as either true or false aneurysms. True ones are a full-thickness, segmental dilation of a vessel with at least a 50% increase in diameter compared with the expected normal diameter [4]. As a result of carotid trauma or prior carotid dissection, when a segmental disruption of the arterial wall happens, false aneurysm or pseudoaneurysm develop and they usually represent a hematoma that has maintained a persistent connection with the arterial lumen. 

True aneurysm to pseudoaneurysm proportion varies. A review from the Mayo Clinic over a 15-year study period (1998 to 2012) which included 141 carotid aneurysms, reported that 82 percent were pseudoaneurysms and 18 percent were true aneurysms [7]. More men were affected by pseudoaneurysm according to reports, whereas for true aneurysm, the opposite was true [1, 7]. The described case is unusual since the patient presented with a true aneurysm and also according to gender distribution.

The most frequent site reported for true aneurysm in the literature reviews is the bifurcation of the carotid artery or proximal internal carotid artery [2, 8]. In the Mayo Clinic review, 81% involved the internal carotid, 8% the common carotid, 10% the bifurcation, and 1% the external carotid [7]. Bilateral aneurysms can also occur [7, 8]. Comparing to these reports the presented case had also an unusual site of aneurysm at distal part of ICA. The factors leading to each type of aneurysm differ. The most common etiology of extracranial carotid artery aneurysm is atherosclerosis (34 to 42%) followed by trauma (35 to 51%) and pseudoaneurysm at prior endarterectomy sites (26 to 57%) [7]. According to other review of 42 patients with ECA, 50% were due to atherosclerosis, 30% were pseudoaneurysms, and 12% were related to trauma [9]. In another report that included 48 patients, 71% were atherosclerotic, 25% pseudoaneurysm, and 4% related to infection [10]. Blunt or penetrating cerebrovascular injury can also lead to extracranial carotid artery aneurysm, which can present acutely or in a delayed manner years after the sinciting event; a time interval between 1 and 20 years has been reported [11, 12].

A variety of other pathologies that are known to weaken the integrity of the arterial wall can also lead to true aneurysm formation, although ECA as manifestation of connective tissue disease is rare [13, 14]. The presented case did not have neither any risk factor for atherosclerosis nor any clue for connective tissue disorders. Open surgical intervention remains the primary treatment choice for carotid artery aneurysms [7]. Open surgical options include carotid artery ligation with or without bypass, and aneurysm excision with reconstruction.  

Carotid artery ligation without bypass reported to be associated with high rates of morbidity (up to 25%) and mortality (20%) [6, 15], and usually is performed for cases where distal control cannot be obtained due to anatomic reasons or for difficult-to-control hemorrhage as with carotid aneurysm rupture, as it happens in the current reported case but without mortality or any significant morbidity. Carotid ligation alone without reconstruction also may be necessary for some mycotic aneurysms following excision of the aneurysm and debridement to healthy tissues [1, 5, 12].

 In conclusion, the aneurysms of the extracranial segment of the internal carotid artery are no common and could be misdiagnosed with other lesion of the cervical region. Thus precise preoperative assessment and evaluation with different modalities should be performed to avoid fatal complications. Surgery is a safe and effective method in experienced hands for repair of such aneurysms. 

## Conflict of Interest:

There isn’t any conflict of interest to be declared regarding the manuscript.
